# Herbal Medicinal Products from *Passiflora* for Anxiety: An Unexploited Potential

**DOI:** 10.1155/2020/6598434

**Published:** 2020-07-20

**Authors:** Lyca R. da Fonseca, Rafaele de A. Rodrigues, Aline de S. Ramos, Jefferson D. da Cruz, José Luiz P. Ferreira, Jefferson Rocha de A. Silva, Ana Claudia F. Amaral

**Affiliations:** ^1^Laboratory of Medicinal Plants and Derivatives, Department of Chemistry of Natural Products, Farmanguinhos, Fiocruz, Rio de Janeiro 21041-250, Brazil; ^2^Professional Postgraduate Program in Management, Research and Development in Pharmaceutical Industry, Farmanguinhos, Fiocruz, Rio de Janeiro 21041-250, Brazil; ^3^Academic Postgraduate Program in Translational Research in Drugs and Medicines, Farmanguinhos, Fiocruz, Rio de Janeiro 21041-250, Brazil; ^4^Faculty of Pharmacy, Fluminense Federal University, Niterói 24241-002, Brazil; ^5^Laboratory of Chromatography, Department of Chemistry, Federal University of Amazonas, Manaus 69077-000, Brazil

## Abstract

Herbal medicines containing *Passiflora* species have been widely used to treat anxiety since ancient times. The species *Passiflora incarnata* L. is included in many Pharmacopoeias, and it is the most used species in food, cosmetic, and pharmaceutical industries. However, there are around 600 species of the genus *Passiflora* and probably other species that can be used safely. Thus, this article was based on a search into the uses of the main species of the genus *Passiflora* with anxiolytic activity and its main secondary metabolites and some pharmacological studies, patents, and registered products containing *Passiflora.* Furthermore, the Brazilian Regulatory Health Agency Datavisa, Medicines and Healthcare Products Regulatory Agency of the United Kingdom, and the European Medicines Agency websites were consulted. The results showed that *Passiflora* species have health benefits but clinical trials are still scarce. The complexity of *Passiflora* extracts creates challenges for the development of herbal medicines. *P. incarnata* is the most studied species of the genus and the most used in natural anxiolytic herbal medicine formulations. However, there are hundreds of *Passiflora* species potentially useful for medicinal and nutraceutical purposes that are still little explored.

## 1. Introduction

The contemporaneous tense lifestyle is responsible for the wide use of anxiolytic substances, mainly those of the benzodiazepine group. This class of synthetic drugs is one of the most prescribed antianxiety drugs in the world to treat stress and anxiety. However, many risks are associated to their use, such as dependence, abstinence syndrome, and the reduction of motor reflexes. Therefore, the use and acceptance of herbal medicines and nutraceuticals for these purposes, including *Passiflora* species, have been growing among doctors and patients [1]. *In vitro* and *in vivo* studies are needed to establish the bioequivalence of herbal formulations. In the case of *Passiflora* formulations, the standardization can be based on the potency in animal models, such as the open arm plus-maze using rats or mice. After the isolation of the substances responsible for the anxiolytic activity, the pharmaceutical equivalence can be applied [2].

Many countries have laws regulating herbal medicinal products. Germany, that is considered the world's largest market for these products [3], defines herbal medicinal products as “medicinal products that exclusively contain, as active substances, one or more herbal substances, one or more herbal preparations, or one or more such herbal substances in combination with one or more such herbal preparations, according to the German Drug Law [4].” Estimates suggest that Germany spends more than 2 billion dollars on herbal medicinal products each year [3].

The Directive 2001/83/EC [5] and Directive 2004/24/EC [6] of the European Parliament of the Council of the European Union are codes for medicinal products and traditional herbal medicinal products for human use. The code defines herbal medicinal products as any medicinal product exclusively containing, as active ingredients, one or more herbal substances (whole or fragmented plants) or herbal preparations (tinctures, extracts, essential oils, etc). In the same code, traditional herbal medicinal products have simplified registration, because they can be used without prescription or monitoring treatment and the products are not harmful when used according to the instructions of the manufacture. They may be administered orally, topically, or by inhalation. The pharmacological effects or efficacy are based on long experience (at least 30 years, including at least 15 years within the European Community) but the safety of the product must be ensured, and the quality must be verified according to the European Pharmacopoeia monographs, or those in the Pharmacopoeia of a Member State [7].

The regulatory requirements for the registration of herbal medicines in Brazil are similar to those in Europe. In Brazil, these preparations are classified as herbal medicines if they are obtained only from active raw vegetal material, of which the efficacy and safety have been validated through ethnopharmacological surveys, scientific and technical documentation, or clinical evidence [8]. These products may be registered and marketed as traditional herbal medicine, if evidence of their safe and effective use has been published in scientific and technical literature, and there is evidence of continuous safe use for a minimum period of thirty years. Furthermore, they may not be used for any serious diseases; they may not contain materials in toxic concentrations; and they should not be administered by the ophthalmic or intravenous routes. The safety and efficacy of herbal medicines are based on clinical evidence, and they are characterized by consistent quality [9]. Any registrations or marketing authorizations in other countries, and the reasons for refusal of registration or marketing authorization must be stated [3]. Traditional herbal medicinal products are designed for use without the supervision of a physician in terms of diagnosis, prescription, or monitoring.

Arifin and coworkers [10] list and discuss several challenges that impact the development of a herbal medicinal product. Normally, the difficulties present in the production of a herbal medicine are related to the isolation of the active ingredient(s) and its pharmacological evidence, safety, and therapeutic efficacy. The complex nature of plants creates difficulties, and consequently, there are challenges for the development of herbal medicinal products, which is not a quick and simple process. Plant species have various phytochemicals with different characteristics, e.g., absorption, solubility that affect their bioavailability. Additionally, there are the possible unknown toxic effects, potential harmful interactions of herbal medicinal products and the lack of pharmacokinetic parameters such as gastrointestinal absorption, protein binding, and plasma clearance by the liver and kidneys, which are all important challenges to overcome to be able to produce new medicines from medicinal plants. The quality of herbal products from a technological point of view is ensured for the intermediate and finished products, using adequate production planning, appropriate production processes, and strict control of the raw materials from the plant species used. All of these cares must ultimately result in products with constant quality, efficacy, and security.

This article was based on a literature search into the widespread use of the main species of the genus *Passiflora* and their main secondary metabolites to treat anxiety. The search covered pharmacological studies, patents, and registered products that were focused on the promising Brazilian market. This report about *Passiflora* herbal medicinal products comprises eight sections: *Passiflora* genus (Passifloraceae); chemical constituents; mechanism of anxiolytic action; *Passiflora* extract: standardization and chemical marker; pharmacology, pharmacokinetics and safety; patents and intellectual property in Brazil; registered medicines containing *Passiflora* in Brazil; and patents and registered products containing *Passiflora* outside Brazil.

## 2. Methods

Empirical searches were conducted via the databases Scopus® (http://www.scopus.com) [11], Scirus (http://www.scirus.com) [12], SciFinder® (http://scifinder.cas.org/) [13], Google Scholar (https://scholar.google.com.br/) [14], as well as periodicals using the following three keywords: ‘*Passiflora incarnata*', ‘*Passiflora edulis*', and ‘*Passiflora alata*'. Other keywords used with the genus *Passiflora* were extraction, pharmacophores, ADMET, flavonoids, and formulation. A search for clinical trials using the keywords ‘anxiety' and ‘Passiflora' were performed in the database ClinicalTrials.gov (https://clinicaltrials.gov), of the National Institutes of Health (NIH), an agency of the U.S. Department of Health and Human Services [15]. All of the information about the plants, their past and present therapeutic uses, and data about marketing of herbal medicines in Brazil and outside Brazil, including species of *Passiflora* in combination with other active ingredients, were taken directly from selected papers and searched in the following websites: the National Institute of Industrial Property (Instituto Nacional de Propriedade Industrial, INPI), https://gru.inpi.gov.br/pePI/servlet/PatenteServletController [16]; National Health Regulatory Agency (Agência Nacional de Vigilância Sanitária, ANVISA), https://consultas.anvisa.gov.br/#/medicamentos/, an agency of the Ministry of Health [17]; Trade-Related Aspects of Intellectual Property Rights (TRIPS Agreement) [18], the Brazilian Pharmacopoeia [19], Medicines and Healthcare Products Regulatory Agency (MHRA) of the United Kingdom [20], and European Medicines Agency [21].

The graphs and tables were constructed using Excel 2013.

### 2.1. *Passiflora* Genus (Passifloraceae)

According Muschner and colleagues [22], the Passifloraceae family is divided into two tribes: Paropsieae, with six genera, is found throughout Europe, Africa, and Madagascar, and Passiflorieae, with 14 genera, is found in the Americas and in Europe. The genus *Passiflora*, belonging to the Passifloraceae family, contains approximately 530 species and 400 artificial hybrids [23] or, according to other authors, more than 600 species [22, 24, 25]. At least 140 are native to Brazil, and 70 of them produce edible fruits. Brazil is the largest producer of *Passiflora* fruits in the world, with a reported annual production of 923,035t [26]. It is popularly known as passion fruit in English and “maracujazeiro” in Portuguese [27, 28]. Besides the nutritional use of the fruits, the passion flower is cultivated as an ornamental plant and used in the cosmetic and pharmaceutical industries. The sedative properties of its leaves and hypoglycemic activity of the flour produced from the epicarp of the fruit have already been described [26, 29]. Species of *Passiflora* are rich in flavonoids, which have anxiolytic activity, among other activities. *P. incarnata* L. is the most widely studied species, due to its chemical composition and its pharmacological effects [30]. However, biological activities similar to those described for *P. incarnata* have been reported for other Brazilian species of the same genus, providing an alternative source for the formulation of herbal medicines.

Despite the many different species of *Passiflora*, only two are reported in the Brazilian Pharmacopoeia, 6th Edition [19], *Passiflora edulis* Sims and *Passiflora alata* Curtis. In Brazil, herbal medicines are prepared using the native species *P. alata*, *P. edulis* f. *flavicarpa* [31], and the exotic *P. incarnata*. *P. incarnata* is the most widely studied from a pharmacological point of view and it is present in the official pharmaceutical codes of different countries, including the British Herbal Pharmacopoeia, the United States Homoeopathic Pharmacopoeia, the Homoeopathic Pharmacopoeia of India, the Pharmacopoeia Helvetica, the Egyptian, French, German and Swiss Pharmacopoeias, and the British Herbal Compendium [27, 31, 32].

There have been numerous taxonomical, pharmacological, and toxicological studies on *P. edulis* and *P. alata*, but for other species native to Brazil, although cataloged for a long time, there is still a lack of studies on their potential uses in food, cosmetics, and pharmaceuticals [32]. Different activities have been reported for *P. incarnata*, especially in the pharmaceutical field. These include its action on the central nervous system for the treatment of anxiety disorders and neuropsychic symptoms of menopause, such as pain, anxiety, sexual dysfunction, and sleep disorders.

### 2.2. Chemical Constituents

In phytochemical studies, some papers have reported that the flavonoids and alkaloids of *Passiflora* may be related to its anxiolytic properties [33–35]. Some variability of flavonoids may occur within this botanical genus throughout the year, and there are different methods to increase the concentration of these metabolites in the leaves [36]. The literature indicates that some species of *Passiflora* have differences in their flavonoid C-glycosides contents, and these differences are relevant to the characterization of their specific origins. For example, isovitexin is found in higher amounts in *P. incarnata* [33, 37], *P. alata* [38], and *P. edulis* var. *flavicarpa* and *P. edulis* var. *edulis* [38], although it was not detected in *P. edulis* var. *edulis* by Zucolotto and colleagues [39]. Moreover, orientin and vitexin are found in small amounts in the leaves of *P. incarnata*, *P. alata*, and *P. edulis* [37–40]. Furthermore, iso-orientin is one of the major flavonoids in *P. incarnata* and *P. edulis* var. *flavicarpa*, but it is only detected in small concentrations in *P. alata* [37–39]. Additionally some C-glycosides were identified in just one species, such as luteolin-6-C-chinovoside and luteolin-6-C-fucoside in *P. edulis* [24, 33] and 2″-xylosylvitexin in *P. alata* [33]. A comparative metabolite profiling and fingerprinting of the genus *Passiflora* leaves were reported using a multiplex approach of UPLC-MS and NMR analyzed by chemometric tools [41]. Thin layer chromatography can also be used in order to differentiate species [42]. Recently, new components were isolated and identified in the fruit peel of *Passiflora edulis*, including five flavonoids [43, 44].

Based on studies that indicate the presence of flavonoids in extracts of *Passiflora* and their pharmacological properties, a total flavonoid analysis has been proposed for the preliminary evaluation of this plant species [42, 45–49]. Furthermore, the C-glycosylated flavonoid may represent a particularly important chemical marker for the quality control of herbal medicines, enabling the identification of different species of *Passiflora* [46, 50, 51].

**Figure 1:**
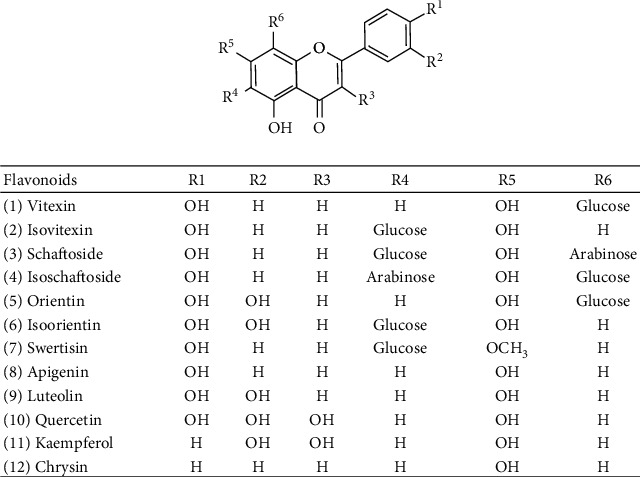
Flavonoids of *Passiflora incarnata*.

C-glycosylated flavonoids found in *P. incarnata* consist mainly of a glucose group directly linked to the aromatic nucleus only in positions 6 and 8 of the flavonoid core [52]. The aqueous extract is phytochemically characterized by a set of C-glycoside flavonoids such as vitexin (1), isovitexin (2), schaftoside (3), isoschaftoside (4), orientin (5), iso-orientin (6), and swertisin (7) [53, 54]. Moreover, the free flavonoids apigenin (8), luteolin (9), quercetin (10), kaempferol (11), and chrysin (12) are also found [33].  Figure 1 illustrates the chemical structures of the flavonoids of *P. incarnata*.

**Figure 2:**
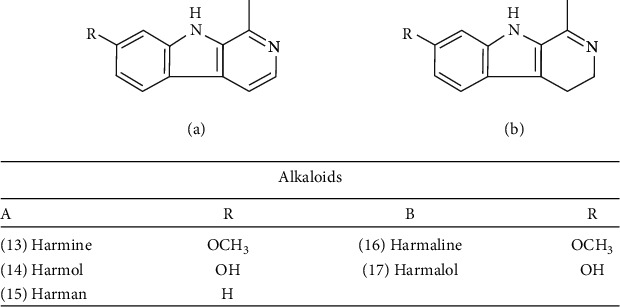
Alkaloids of *Passiflora incarnata*.

The alkaloids present in *Passiflora* are of the indole type (*β*-carbolines), which are the second major known group of alkaloids [54]. Some of them have value in medicine as tranquilizers and for the treatment of hypertension [55]. *P. incarnata* is the most widely studied species containing alkaloids [24]. In studies carried out in the 1960s, harmine (13), harmol (14), harmaline (15), harmalol (16), and harman (17) were detected (Figure 2) [56]. Although the presence of traces of these alkaloids was confirmed [24], they were undetectable in most commercial materials [57]. Figure 2 shows the principal alkaloids mentioned above.

### 2.3. Mechanism of Anxiolytic Action

Events related to depression and anxiety, among others in the central nervous system, refer to the balance between chemical excitation and inhibition [58]. One of the mechanisms involves the *γ*-aminobutyric acid (GABA) system via binding to the benzodiazepine-site of the GABA type A receptor (GABA_A_), which can be modulated by allosteric agents, for example, benzodiazepines, which promote the regulation of the chloride flow through the ion channel complex [59]. The possibility of elaborating or identifying new drugs with chemical characteristics of benzodiazepines for the treatment of anxiety led to the formulation of pharmacophoric models, and the model designed by Cook is the reference in this field of study. The pharmacophoric model designed by Cook describes a lipophilic region, two hydrogen bond donor sites (HB1 and HB2), and a hydrogen bond acceptor site (A2) [59, 60]. These characteristics are consistent with the chemical structure of flavonoids, mainly the two hydrogen bond-donating sites that are related to ether oxygen and a carbonyl group present in flavones, and the aromatic rings A and B corresponding to the lipophilic regions L1 and L2, respectively [61]. In order to elucidate some incongruencies related to actions not predicted by this model in the study of 6-methyl-3′-nitroflavone [62], a new pharmacophoric model was designed. In this new model, in addition to the regions described by Cook's model, there are two sites classified as S1 and S2 relative to electrostatic interaction of substituents in 6-position of flavone [61, 63]. Additional studies revealed two new regions of steric repulsive around the 4′- and 5′-regions of the B-ring [61, 64].

The chemical constituents responsible for the anxiolytic activity of *Passiflora* are not yet fully understood but the majority of published works suggest that phenolic substances, especially the flavonoid class, are related to this property [65–68]. The flavonoids identified in *P. incarnata* (Figure 1) probably act according to the pharmacophoric models described. The mechanism of action is probably related to the modulation of the *γ*-aminobutyric acid (GABA) system, because *Passiflora* flavonoids are partial agonists of GABA_A_ receptors and inhibit the uptake of [3H]-GABA into rat cortical synaptosomes [30, 67, 69, 70]. The review article written by Wasowski and Marder [58] describes flavonoids as GABA_A_ receptor ligands, including apigenin and chrysin that bind to the benzodiazepine-binding site of the GABA_A_ receptor, exhibiting anxiolytic activity, without evidencing sedation and muscle relaxant effects [58, 71]. *In vitro* assays demonstrated that the flavonoid chrysin (5,7-dihydroxyflavone) has affinity for the benzodiazepine receptors and that it increases pentobarbital-induced hypnosis and reduces locomotor activity in mice after intraperitoneal administration of 30.0 mg/kg body weight (bw) [57]. This is reinforced by studies that indicate that the pre-administration of flumazenil, an antagonist of GABA_A_ receptor, attenuates the anxiolytic effects of passionflower like diazepam. However, this is possible not the unique mechanism involved [30].

In one study, kaempferol exhibited a very low affinity for the benzodiazepine-binding site and was devoid of anxiolytic actions by the intraperitoneal route [58]. However, in another study to evaluate the anxiolytic activity of the flavonols, kaempferol, quercetin, and myricetin, in the elevated plus-maze after oral and intraperitoneal (i.p.) administration in mice, only kaempferol and quercetin were active after oral administration, although they were inactive via i.p. [72].

The harman alkaloids found in *P. incarnata* which belong to the *β*-carboline class have different structural characteristics that interact with benzodiazepine receptors. This class of alkaloids attracted the attention of Braestrup and colleagues [73] during studies with endogenous ligands for benzodiazepine receptors after the detection of *β*-carboline-3-carboxylic acid ethyl ester in normal human urine. This discovery was important because it was foreshadowing the synthesis of several derivatives of *β*-carboline compounds with important biological activity [74]. In addition, research involving *β*-carboline compounds has led to conceptual and experimental considerations, covering the role of ligands in the GABA_A_ receptor modulation process in a spectrum that ranges from full agonists to full inverse agonists [75]. Furthermore, *β*-carboline alkaloids are consistent with the pharmacophoric model developed by Cook and coworkers [59, 60], enabling the recognition of a hydrogen bond acceptor (A2) site in the NH group, a hydrogen bond donor (H1) in N(2) [75], in addition to the positions in C6 and C3 framed in the lipid domain (LDi) [76].

### 2.4. *Passiflora* Extract: Standardization and Chemical Marker

The marketing of extracts obtained from plant drugs is advantageous for the industry in several ways, as it reduces the steps involved in the manufacturing of herbal medicine products, it minimizes waste production, and it simplifies analytical quality control. However, an important point for evaluation is the standardization of this extract obtained commercially. Standardization of raw plant materials can be done by the chemical quantification of substances or class of substances related to the therapeutic effects, or through the presence of chemical marker substances of the species. The most abundant substances in the leaves of this species belong to the class of flavonoids, especially C-glycosides, which are directly related to its pharmacological activities [24].

According to the current Brazilian regulations for herbal medicines with simplified registration, Normative Rule no. 02 determines that the main chemical marker of the *P. incarnata* extract is vitexin, which is considered to be the active ingredient of its aqueous extract. The range of a daily dosage varies from 30 mg to 120 mg of total flavonoids expressed as vitexin in an herbal medicine made from *Passiflora* [77]. According to the Assessment Report on *P. incarnata*, elaborated by the European Medicines Agency (EMA) that is affiliated to the European Union, the herbal extract prepared with hydroalcohol solution or acetone solution may contain at least 2.0% of flavonoids expressed as vitexin, and the daily dose recommended corresponds in most cases to about 20–30 mg of total flavonoids [21]. The Brazilian Pharmacopoeia does not contain any information about the minimum flavonoid contents in extracts, but British and French Pharmacopoeias recommend at least 1.5% of flavonoids expressed as vitexin [78].

The continuous growing demand for plant derived therapeutic molecules obtained in a sustainable and eco-friendly manner favors biotechnological production leading to the production of plants with increased levels of desirable compounds with potential biological activity. Similarly, the development of innovative extraction techniques to obtain phytoconstituents that can be used in modern medicine is another approach that must be taken into account. On this point, accelerated solvent extraction can be used to obtain extracts enriched in polyphenolic compounds from different species of *Passiflora* sp. [38]. This extraction method offers many advantages such as good reproducibility and shorter extraction time, the possibility to adjust extraction temperature, and purification of extracts online. Fierascu and colleagues [79] suggest some techniques that may be combined and applied in the development of products based on *Passiflora*.

### 2.5. Pharmacology, Pharmacokinetics, and Safety

Information on the absorption, distribution, metabolism, elimination, and toxicity (ADMET) of a drug in the human body is of great importance for the characterization of a drug formulation regarding the treatment of a certain disease and its safety for the patient [80, 81]. Knowledge of ADMET properties is usual for synthetic drugs; however, when the universe of substances present is expanded, such as in the case of products from plant species, there is a reduced level of information about these properties [80–82].

The reasons for this gap are the complexity of a formulation based on natural products, which generally includes a high number of substances in its composition that can suffer qualitative and quantitative variation of these constituents due to many factors, such as plant harvest and climatic factors [80, 81]. Currently, it is possible to apply standardized methods for the preparation of herbal medicines with the chemical composition controlled. However, the identification of the active substance is usually difficult, and this fact can compromise the knowledge of the ADMET properties in the body [80, 82].

The alternative to deal with these peculiarities related to herbal medicines is the determination of ADMET properties of pure constituents of the formulation, through *in vitro* tests, for example, Caco-2 cells, human liver microsomes, and cultured human hepatocytes [80]. In this context, the ADMET properties of the flavonoids iso-orientin, isovitexin, luteolin, apigenin, and kaempferol present in *P. incarnata* have been described in the literature [80, 83, 84].

Absorption processes are evaluated by blood-brain barrier (BBB) penetration and human intestinal absorption (HIA) methods that demonstrated high absorption through human intestine and side effects on absorption through BBB for iso-orientin, isovitexin, and luteolin. Apigenin and kaempferol showed positive values for both BBB and HIA [80, 83, 85]. The importance of a substance crossing the BBB is partially related to the required biological activity, and this is based on structural and physicochemical characteristics, such as lipophilicity, molecular size, desolvation potential, pKa-to-charge ratio, and hydrogen bond [80, 86, 87].

In metabolic process, CYP450 is an isoenzyme that metabolizes several substances, including, carcinogens, fatty acids, bile acids, and steroids, which evidences its importance in the characterization of the metabolic properties of various substances. Among the previously mentioned flavonoids, only iso-orientin and isovitexin, have low CYP inhibitory promiscuity. However, all of them were nonsubstrate for all CYP450 substrates (2C9, 2D6), and most of them inhibited most of the CYP450 inhibitors (2C9 and 2D6) [80, 83, 85]. The inhibition of CYP450 isoforms is closely related to the metabolism of a substance and can lead to increased toxicity [85]. Aspects related to toxicity were measured based on biodegradability, Ames toxicity, and carcinogenicity. In general, the toxicological profile of the flavonoids indicates that they are not carcinogenic and, with the exception of iso-orientin and isovitexin, demonstrated to be negative for Ames toxicity [80, 83, 85].

Within a broader context, the flavonoids present in *P. incarnata* have a good ADMET profile and, considering an herbal formulation containing these substances, i.e., good absorption, metabolism, distribution, elimination, and low toxicity.

Given these premises, knowledge about pharmacokinetic parameters is an extremely important aspect for the development of any oral formulation of *Passiflora* extracts. The water solubility of flavone molecules, such as for most of the flavonoids, is limited, and it is 0.1 g/L for chrysin. Flavonoid aglycones generally tend to have good membrane permeability, whereas flavonoid glycosides have poor membrane permeability, related to their ability to form hydrogen bonds, and consequently, aglycones are absorbed better and faster. Pure chrysin seems to be predominantly absorbed by passive diffusion. All flavones, including those with an unsubstituted B ring, are rapidly metabolized in the small intestine and then supplemented with biotransforms in the liver, while those that are not absorbed are affected by the additional metabolism of bacterial enzymes in the colon. The prolonged metabolism of the first pass of flavonoids, mainly by glucuronidation and sulfation, proved to be the main reason for their low bioavailability. Glucuronidation of flavones by UDP-glucuronosyltransferases (UGT) 1A1 represents together with sulfation by sulfotransferase (SULT) 1A3 and 1A1 the most important metabolic pathway of these natural molecules when ingested by humans. Chrysin is predominantly glucuronidated by UGT1A1, having preeminence over sulfation [88, 89].

General data of flavonoid excretion indicate that most of these glucuronides are subject to biliary excretion, with little urinary contribution. In the case of chrysin, only about 3% of the dose administered is excreted through the kidneys. However, most of the glucuronides tend to be pharmacologically inactive, although notable exceptions are known [88, 89].

The third volume of the World Health Organization monographs on selected medicinal plants contains the monograph of *P. incarnata* [57]. In this monograph, experimental pharmacology studies related to central nervous system depressant activity in mice are described. The aqueous or hydroalcohol extracts (30–40% of ethanol) of the aerial parts reduces spontaneous motor activity, increased pentobarbital-induced sleeping time, and potentiated pentobarbital-induced sleeping time after intraperitoneal or intragastric administration. However, the aqueous extract seems to be more active than the hydroalcohol extract, since some activity is observed with the aqueous extract at 25.0 mg/kg (bw), while hydroalcohol extracts only have some activity at higher concentrations (50.0 mg/kg or over) [57].

The aqueous extract is toxic to mice at 900.0 mg/kg bw i.p. and the oral median lethal dose (LD_50_) of a 30% ethanol extract in mice was 37.0 ml/kg bw [57]. Other nonclinical information indicates that the acute and repeated dose toxicity is low, and the LD_50_ of the hydroalcoholic extract in mice and rats is higher than 15 g/kg orally and higher than 3 g/kg i.p. [21]. There is no evidence of genotoxicity, but *P. incarnata* is contraindicated during pregnancy because it seems to stimulate uterine contractions in animal models. It may cause drowsiness and therefore care is recommended for activities that require attention. Some adverse events that are very rare include hypersensitivity, ventricular tachycardia, nausea, vomiting, drowsiness and prolonged QT interval, thrombocytopenia, left ventricular failure, ventricular fibrillation, hepatic function abnormal, arrhythmia, tremor, agitation, and withdrawal syndrome. However, no correlation of serious adverse events and *P. incarnata* intake has been established. There are no reports regarding safety for nursing mothers and children, and the use of *P. incarnata* in these cases should be monitored by a physician [21, 57]. Furthermore, the concomitant use of *P. incarnata* and sedatives such as benzodiazepines, zolpidem or barbiturates, alcohol, and clonidine is not recommended due to possible pharmacokinetic interactions [90].

Clinical research studies involving the use of *Passiflora* to treat anxiety were searched for in the ClinicalTrials.gov, a database maintained by the National Institutes of Health (NIH), which is an agency of the U.S. Department of Health and Human Services [15]. This database registers private and public clinical studies from throughout the world. There are six research studies registered that are listed in Table 1, all of them with the species *P. incarnata*. Four studies have been concluded or terminated, and two are unknown. The results concerning the effectiveness or the reasons for discontinuation were not informed. Only one of the studies was conducted with monopreparations of *Passiflora*. The concomitant use of plant species in clinical trials makes it difficult to attribute any effect specifically to *P incarnata*.

In the literature, there are few relevant clinical trials available for monopreparations of *P. incarnata* to support its use to treat anxiety, and the published studies should be considered preliminary and only suggestive of its effectiveness. The most complete study is described by Mori and colleagues [91], which was evaluated by The Cochrane Collaboration [92] and EMA [21]. According to these evaluations, the multicentre double-blind study performed in Japan over a 4-week period was adequately randomized, and the Jadad score was 5. In the study, 63 patients received *P. incarnata* extract (Passiflamin) and 71 patients received mexazolam. The initial doses were 90 mg of Passiflamin or 1.5 mg of mexazolam, which were further doubled. Although mexazolam was more effective, the efficacy of Passiflamin was significant to treat anxiety, tenseness, and irritation.

A small study with 36 patients with general anxiety over a 28-day period demonstrated that the use of passionflower extract (45 drops/day) is as effective as oxazepam (30 mg/day) [93]. The same research group evaluated the effect of *P. incarnata* extract (60 drops) associated with clonidine (0.8 mg) in opiate addicts undergoing withdrawal [94]. In the study with 65 patients, the authors concluded that the daily administration of the combination over 14 days was better than only clonidine at 0.8 mg/day.

Movafegh and colleagues [95] conducted a clinical study with 60 patients that were premedicated 90 min before a surgery. According to the numerical rating scale to assess anxiety and sedation, the anxiety scores of the group that received a 500 mg tablet containing 1.01 mg benzoflavone was lower than the anxiety scores of the group that received placebo, without inducing sedation. Another study involving preoperative anxiety was conducted with 60 patients 30 minutes before spinal anesthesia [96]. Patients received 5 mL of an aqueous solution containing 700 mg of an aqueous extract of *P. incarnata* containing 2.8 mg benzoflavone or 5 mL of mineral water. A small difference was observed between the two groups in the State Anxiety Inventory score, without sedation. Dantas and colleagues [97] studied the effect of *P. incarnata* (pill containing 260 mg) or midazolam (pill containing 15 mg) in forty volunteers for the control of anxiety before undergoing bilateral extraction of their mandibular third molar. Over 70% of the patients reported that they felt quiet or a little anxious with both treatments, and no significant differences were observed between the groups. However, 20% of the participants reported amnesia with midazolam. The authors found that *P. incarnata* showed an anxiolytic effect similar to midazolam.

The effect of *P. incarnata* on sleep quality was studied in a double-blind study with 41 volunteers [98]. Over a 7-day period, the volunteers received placebo (dried parsley infusion) or a *P. incarnata* infusion prepared with teabags containing 2 g of aerial parts and 250 mL of boiling water during 10 min. The overnight polysomnography on the last night of the treatment indicated a significant increase of 5.2% of the sleep quality, but no significant effect on total sleep time and nocturnal awakenings.

It is important to highlight the lack of detail regarding the composition of *P. incarnata* extracts or even the composition of the formulations. Nonclinical studies suggest that aqueous extracts are more active than the 40% ethanol extracts, which indicates that small variations in chemical composition can be determinants in activity. The standardization of vegetable raw materials is of great importance in this area. The complex chemical composition of *Passiflora* extracts and the lack of knowledge of the pharmacological activity of each component increase the challenges, due to the difficulties in determining the most appropriate chemical marker in the standardization of the extract and in stability studies that any new studies should be directed to, for the development of formulations containing *Passiflora*. Other important considerations regarding the clinical trials presented here were previously mentioned by Miroddi and colleagues [30] who reviewed ethnopharmacological and clinical trials related to *P. incarnata*. The studies were performed with a small number of participants, and some studies had inadequate comparison between the placebo and the medications recommended for anxiety and gave little importance to the use of *Passiflora* found in some results. Thus, the clinical trials reported are pilot studies and are not enough to recognize the medicinal use of *P. incarnata* as being well-established. Moreover, the actual recommendations are still based on its long-standing use to relieve mild symptoms of mental stress. Thus, *P. incarnata* and other species of the genus offer a wide field for research that is still little explored, which should involve interdisciplinary and interinstitutional partnerships, covering the stages of phytochemical studies for standardization and developments in herbal medicines, along with nonclinical and clinical studies.

### 2.6. Patents and Intellectual Property in Brazil

The Agreement on Trade-Related Aspects of Intellectual Property Rights (TRIPS Agreement), of which Brazil is a signatory, established the current standards of intellectual property protection worldwide. It came into force on January 1, 1995 and is mandatory for all member countries of the World Trade Organization [18]. The TRIPS Agreement establishes minimum standards under international law related to patents, including standards for drugs. The members of the World Trade Organization (WTO) agreed on the development and implementation of patenting laws according to common patterns. These models state, among other things that patents should be granted for a minimum of twenty years, that patents may be granted for products and processes and that information for drug tests must be protected against “unfair commercial use”.

In Brazil, the Law of Intellectual Property [99] excludes from patentability all plants and animals, or parts thereof, except for transgenic microorganisms with the requirements of patentability (novelty, inventive step, and industrial application) and those that do not constitute mere discoveries. Thus, Brazilian law does not allow the patenting of plant extracts. However, processes for obtaining extracts or isolated active ingredients from plants, pharmaceutical compositions and their methods of preparation, and other uses of the same products obtained from plants, are patentable. In this way, the growing herbal market aligned to government policies to promote regularization of medicinal plants and their derivatives [100], and the innovation for the development of herbal medicines justifies the product patent protection [101].

On that basis, the keyword <Passiflora> was selected as a search term, to search in the website of the National Institute of Industrial Property (Instituto Nacional de Propriedade Industrial, INPI). The results returned a miscellany of food products, cosmetics, packaging materials, manufacturing processes for simple syrups for dietary use, herbal syrups, and formulations containing bronchodilators, among others. The results of twenty-eight patents are shown in Table 2. Furthermore, there are recent patent files and they are concerned with obtaining new extracts, production processes, and analytical techniques.

### 2.7. Registered Products Containing *Passiflora* in Brazil

In Brazil, sanitary control of medicinal plants and herbal medicines is done through the “Agência Nacional de Vigilância Sanitária” (ANVISA—National Health Regulatory Agency), an agency of the Ministry of Health, which has the role of ensuring the health safety of products and services [102]. One of the actions taken by ANVISA to secure the health of the population is drug registration, whereby new drugs are evaluated for their safety, efficacy, and quality, before being marketed for use by the population.

The regulatory requirements for herbal medicines cover a range of aspects, from the supplier of the active ingredients through to the final product, produced under strict sanitary standards. The Collegiate Board of Directors Resolution (Resolução da Diretoria Colegiada, RDC) number 26 of May 13, 2014 provides for the registration and notification of traditional herbal products and herbal medicines to register with this agency [5]. The Normative Rule no 2 [77] published on the same date presents the lists of herbal medicines and traditional herbal products with simplified registration. Resolution RDC number 38 of June 18, 2014 [103] and resolution RDC 235 of June 20, 2018 [8] regulate adjustments, changes, extensions, updates, additions, and postregistration notifications of herbal medicines and traditional herbal products. Resolution RDC number 66 of November 26, 2014 establishes the information which has to be shown on the package leaflet of the traditional herbal product [104]. Resolution RDC number 105 of August 31, 2016 requires the presentation of the pesticide residue analysis reports or proof that the medicinal plants cultivated or collected in Brazil come from organic agriculture [105]. According to Resolution RDC 26/2014, both traditional herbal products and herbal medicines must be obtained exclusively from active raw material from plants and cannot include, in their composition, isolated or highly purified active substances [9].With technical and legal support in Brazil, it is possible to develop an innovative herbal medicine with simplified registration.

**Figure 3:**
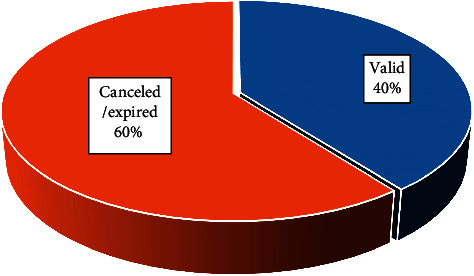
Valid and canceled/expired registrations of *Passiflora*-containing medicines registered in Brazil (ANVISA).

In Brazil, registration with the competent health regulatory agency is required in order to market herbal medicines.  Table 3 shows the products and formulations registered on the Datavisa ANVISA website, including species of *Passiflora* in combination with other active ingredients. The search has included products registered since 1985 since ANVISA does not remove any product from the list in order to maintain the historical record, even after their expired registration. The list includes both regular and revalidated products and those for which registration was canceled or has expired. Although 86 products containing *Passiflora* are registered with ANVISA (Table 3), only 34 medicines are currently valid in Brazil (Figure 3).

**Figure 4:**
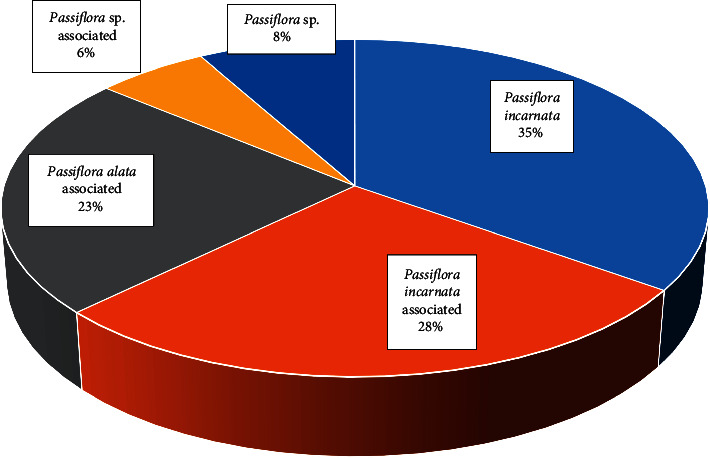
Distribution of *Passiflora* species in ANVISA registered products containing *Passiflora* sp.

The pharmaceutical forms widely marketed and used are capsules, tablets, fluid extract, and tincture, besides infusion, which is a folk preparation. Among the 86 products registered with ANVISA, *Passiflora incarnata* is present in 63% of them. Species of *Passiflora* genus are associated with other plant species in 57% of the products, in which *P. incarnata* predominates (Figure 4). Simple herbal medicines correspond to 43% of the total registered products, in which *P. incarnata* has been used in 30 registered products. The registration for the use of herbal products in association requires proof of the benefits of this composition, and a risk/benefit evaluation [100], as well as the use of *P. alata* as a substituent of *P. incarnata*. Although *P. alata* is registered in the Brazilian Pharmacopoeia [19], this plant species is not listed in the normative rule about simplified registration of herbal medicines [77].

### 2.8. Patents and Registered Products Containing *Passiflora* outside Brazil

Outside Brazil, empirical searches carried out in the database of the United States Patent and Trademark Office, an Agency of the Department of Commerce North American, indicated the existence of 572, 164 and 4 patents when the keywords “passion flower”, “*Passiflora edulis*” and “*Passiflora alata*” were used, respectively [106].

From 2006 to 2013, 27 traditional herbal products containing *Passiflora* were registered with the Medicines and Healthcare Products Regulatory Agency (MHRA) of the United Kingdom. All of them contain extracts of *P. incarnata* in single formulations or in association with other plant species. These associations represent approximately 74% of all the products, and include, in their formulations, species traditionally used to treat anxiety, such as *Valeriana officinalis* (Valerianaceae), *Lactuca virosa* (Asteraceae), *Hypericum perforatum* (Hypericaceae), and *Melissa officinalis* (Lamiaceae) [20].

**Figure 5:**
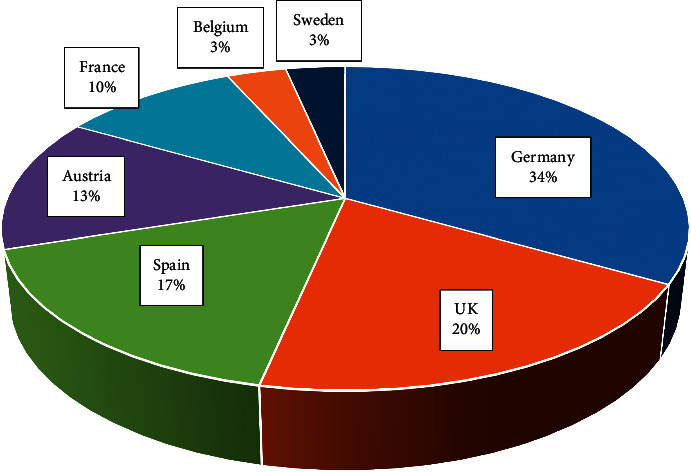
Distribution of preparations containing *Passiflora incarnata* registered in Europe Union, according to the European Medicines Agency [21].

According to the European Medicines Agency, affiliated to the European Union, various herbal preparations containing *P. incarnata* are used in the member countries, with marketing authorization and/or registrations for traditional use. Of the total of thirty preparations registered in Europe, Germany has ten, the UK six, Spain five, Austria four, France three, Belgium one, and Sweden one (Figure 5). The main uses are sachets containing fragmented plant material for use in herbal teas, simple or coated tablets containing dry ethanol extract, hard capsules with powdered or fragmented plant material or dry ethanol extract, instant products containing aqueous extract for the preparation of herbal tea, and liquid products for oral use containing ethanol liquid extract. All these preparations are indicated for the relief of symptoms of mild neurasthenia and for insomnia [21].

## 3. Conclusions

This work deals with the medicinal plants of the genus *Passiflora* that have effects on generalized anxiety disorders. Although it is the genus most used by humans for therapeutic purposes within the Passifloraceae family, especially for the treatment of phenomena related to the anxiety syndrome, it is still restricted to very few plant species. In Brazil, the registered and/or patented products containing extracts of *Passiflora* species only include a small number of taxa, highlighting *P. incarnata* and *P. alata*. Among the 86 registered products in Brazil as “*Passiflora*,” those contain *P. incarnata* in single formulations or in combination with other ingredients represent 63% of the total, suggesting that this species of *Passiflora* genus is the most commonly used in medicines. In other countries, *P. incarnata* is also selected as the main component in natural anxiolytic drug formulations, with a long history of use and inclusion as an official plant drug in various Pharmacopoeias, since its effects are documented. Nevertheless, under the criteria of scientific knowledge about the pharmacological properties of *Passiflora* indicated in nonclinical and clinical studies that might be accompanied by the determination of the chemical composition of the extracts, formulations, and products, other studies are still needed to validate the mechanism of action and recognize the medicinal use of *Passiflora* species as well-established. What other species of *Passiflora* can be used safely in therapy as substituents of those most currently used? What are the criteria for this choice? These questions are important, considering that there are around 600 known species little or unexploited for medicinal purposes and that many of them are mainly distributed in tropical regions, such as Brazil. Therefore, research should be implemented immediately, in order to extend the herbal chain production and increase the commercial value of these products.

## Figures and Tables

**Table 1:** List of clinical trials related to the use of *Passiflora* to treat anxiety registered in NIH.

Trial number	Dosage form	Formulation NAME	Stage	Period	Reference anxiolytic AGENT	Status
NCT00794456	Tablet	Pasalix: *Passiflora incarnata* L., *Crataegus oxyacantha* L., *Salix alba* L.	Phase 3	6 weeks	*Valeriana officinalis* L. (50 mg)	Completed Oct 2013
NCT01178632	Tablet	Passiflorine: *Passiflora incarnata* L., *Crataegus oxyacantha* L., *Salix alba* L.	Phase 3	4 weeks	*Valeriana officinalis* L.	Unknown
NCT00944268	Liquid	*Passiflora incarnata* L., *Crataegus oxyacantha* L., *Salix alba* L.	Phase 3	30 days	None	Unknown
NCT02065843	Capsules	*Passiflora incarnata* L. 100 mg	Phase 2 phase 3	1 h before dental surgery	Midazolam (15 mg)	Completed Sep 2014
NCT03909906	tablets	Euphytose®: 50 mg *Valeriana officinalis* L., 40 mg *Passiflora incarnata* L., 10 mg *Crataegus* sp., 10 mg *Ballota nigra* L.	Not applicable	14 days	None	Completed Dec 2019
NCT00997490	Film-coated sugar-pill	Neurapas balance: hypericum, valerian, passionflower	Phase 3	6 weeks	None	Completed Feb 2003

Source: https://www.clinicaltrials.gov/. Accessed on May 19, 2020.

**Table 2:** List of patents in Brazil related to *Passiflora*.

Publication number	Date	Title
BR 10 2017 027632 5	Dec 20, 2017	Extract and flour of the peel fruit of *Passiflora cincinnata* Mast. (Passifloraceae) for use as hipolipment
BR 10 2017 020281 0	Sep 22, 2017	Preparation of functional goat yogurt with passion fruit (*Passiflora edulis* Sims) and mandacaru (*Cereus jamacaru* DC.) jam
BR 10 2017 020087 6	Sep 20, 2017	Preparation and processing of extra jam with passion flower (*Passiflora edulis* Sims) and mandacaru (*Cereus jamacaru* DC.) flavor
BR 10 2017 010267 0	May 16, 2017	Food supplement based on fruits from Caatinga
BR 10 2017 001368 5	Jan 23, 2017	Antimicrobial potential of extracts from exotic fruit waste (tamarind (*Tamarindus indica* L.), grenadilla (*Passiflora ligularis* Juss.), noni (*Morinda citrifolia* L.), dekopon (*Citrus reticulata* ‘Blanco shiranui'), sapoti (*Manilkara zapota* (L.) P. Royen), tamarillo (*Solanum betaceum* Cav.), and mirtilo (*Vaccinium myrtillus* L.)
BR 10 2016 022442 0	Nov 28, 2016	Formulation and production of symbiotic fermented dairy produced with flaxseed (*Linum usitatissimum* L.) and/or waste from the processing of yellow passion fruit (*Passiflora edulis* var. *flavicarpa* Deg.)
BR 10 2016 014976 2	Jun 24, 2016	Sequential extraction process of bioactive compounds from passion fruit bagasse and the use of its bioactive compounds
BR 10 2015 032464 2	Dec 15, 2015	Formulation for topical use for photoprotection containing *Passiflora cincinnata* Mast.
BR 11 2017 015446 3	Dec 10, 2015	Homoeopathic method for treating colic, intestinal gases, and malaise in children
BR 10 2015 006517 5	Mar 24, 2015	Mixed flour; sweet cookie; and process for producing mixed flour and sweet cookies containing plant extracts of Brazil nuts (*Bertholletia excelsa* Bonpl.), manioc (*Manihot esculenta* Crantz), peach palm (*Bactris gasipaes* Kunth), and passion fruit (*Passiflora edulis* Sims)
BR 10 2015 002902 0	Fev 10, 2015	Lotion based on *Ganoderma lucidum* (Curtis) P. Karst. Spores
BR 10 2015 001385 0	Jan 22, 2015	Composition of products without added sugar based on yellow passion fruit flour
BR 10 2015 002897 0	Jan 16, 2015	Production of antibiotic containing *Passiflora edulis* Sims extract and its use
BR 11 2016 006227 2	Sep 25, 2014	Lipid extract of passion fruit seeds
BR 11 2015 014492 6	Dec 20, 2013	Composition of plant extract for treatment of opious and alcohol abuse
BR 11 2015 014351 2	Dec 18, 2013	Extract of *Passiflora* seeds and cosmetic, dermatological, and nutraceutical compositions comprising the same
BR 10 2012 026263 0	Oct 08, 2012	Composition for hair mask without gluten
BR 10 2012 026281 9	Oct 08, 2012	Composition for hair conditioner without gluten
BR 10 2012 021728 7	Aug 29, 2012	Nanoparticle
BR 10 2012 022381 3	Aug 28, 2012	Compositions for hair oil
BR 10 2012 019583 6	Jul 09, 2012	Compositions for hair mask
PI 0816292-1	Sep 03, 2008	Process for the preparation of an extract of *Passiflora alata* Curtis plant and its use in cosmetic and pharmaceutical compositions
PI 0800544-3	Feb 01, 2008	Analytical technology based on pyrolysis coupled to gas chromatography/mass spectrometry for the characterization and obtaining chemical compounds from extracts of *Passiflora alata* Curtis dried by nebulization
PI 0800705-5	Jan 15, 2008	Flavor capsules for decreasing absorption of lipids and carbohydrates
PI 0602106-9	May 25, 2006	Process for preparing a product based on *Passiflora incarnata* L., pharmaceutical product, and pharmaceutical composition, use, and method of treatment of anxiety and insomnia
PI 0501200-7	Feb 16, 2005	Process for obtaining energy drink based on coconut water and natural passion fruit juice and honey
PI 0501199-0	Feb 16, 2005	Process for obtaining energy drink based on coconut water and natural passion fruit juice
PI 0203346-1	Jun 26, 2002	Composition of synergistic effect with plant active ingredients for higher activities of hydration and tissue regeneration, dermal protection against aggressive external agents, as lotion, cream, gel, or cellulose scarves, presented ready use

Source: https://gru.inpi.gov.br/pePI/servlet/PatenteServletController. Accessed November 26, 2019.

**Table 3:** List of ANVISA registered products containing *Passiflora* sp.

Product/category	Dosage form	Manufacturer Code	Status	Active
A SAÚDE DA MULHER/antispasmodic, anticholinergic, drug combination	Oral solution	57.507.378/0003-65	Valid	Sodium salicilate/*Passiflora alata* Curtis (fluid extract)/‘Agoniada pluméria' (tincture)/bitter orange peel (fluid extract)
A SAÚDE DA MULHER/antispasmodic, anticholinergic, drug combination	Simple dragee/oral solution	45.992.062/0001-65	Canceled	Sodium salicilate/*Passiflora* dry extract/‘Agoniada' dry extract
ACALMIL/herbal compound	Coated tablet	29.333.218/0001-40	Canceled	*Passiflora incarnata* L./*Salix alba* L. (leaves extract)/*Crataegus oxyacantha* L. extract
ACALMIL/herbal compound	Coated tablet	92.265.552/0001-40	Canceled	*Passiflora incarnata* L./*Salix alba* L. (leaves extract)/*Crataegus oxyacantha* L. extract
ALPHALIV/simple Herbal, simple anxiolytic	Oral solution/coated tablet	09.545.589/0001-88	Valid	*Passiflora incarnata L.*
ANSIODORON/potentized preparation	Tablet	56.992.217/0001-80	Valid	*Avena sativa* L./*Passiflora alata* Curtis/*Valeriana officinalis* L
BRONQUIOGEN/herbal compound	Oral solution	51.603.488/0001-82	Canceled	*Caesalpinia ferrea* Mart. /*Roripa nasturtium* Beck/*Mikania glomerata* Spreng./ *Lantana camara* L./*Passiflora alata* Curtis/*Melissa officinalis* L./*Myrospermum erythroxylum* Allem
CALMAN/herbal compound	Coated tablet/oral solution	02.433.631/0001-20	Valid	*Crataegus rhipidophylla* Gand./*Passiflora incarnata* L./*Salix alba* L.
CALMAN/herbal compound	Coated tablet/oral solution	64.088.172/0001-41	Canceled	*Crataegus rhipidophylla* Gand./*Salix alba* L./*Passiflora incarnata* L.
CALMANTE FARMAERVAS/herbal compound	Oral solution	60.565.520/0001-73	Canceled	*Melissa officinalis* L./*Passiflora alata* Curtis */Valeriana officinalis L.*/*Lavandula officnalis Chaix*
CALMAPAX/similar, associated natural products	Coated tablet/oral solution /cropped	33.173.097/0002-74	Canceled	*Passiflora incarnata* L./*Erythrina mulungu* Mart./*Matricaria chamomilla* L./ *Melissa officinalis* L. (only in oral solution)
CALMASYN/simple herbal, simple analytic	Oral solution/coated tablet	17.562.075/0001-69	Valid	*Passiflora incarnata L.*
CALMAVITA/simple herbal	Hard gelatin capsule	56.646.953/0001-86	Canceled	*Passiflora incarnata L.*
CALMI/herbal compound	Oral solution	17.299.140/0001-05	Canceled	*Matricaria chamomilla* L./*Citrus aurantium* L./*Cymbopogon citratus* Stapf/ *Passiflora alata* Curtis
ACALMIL/herbal compound	Syrup	06.597.801/0001-62	Valid	*Passiflora incarnata L.*
CALMIPLAN/herbal compound	Coated tablet	47.100.862/0001-50	Canceled	*Passiflora incarnata* L./*Salix alba* L./*Crataegus rhipidophylla* Gand.
CALMOPLANTAS/simple herbal, simple anxiolytic	Tincture/hard gelatin capsule	79.634.572/0001-82	Valid	*Passiflora incarnata* L./*Salix alba* L./*Crataegus rhipidophylla* Gand.
CALMOXIL/simple herbal, simple anxiolytic	Coated tablet/oral solution	28.643.633/0001-37	Canceled	*Passiflora incarnata L.*
CARDIOPAX GOTAS/Homeopathic association	Oral solution	76.440.528/0001-43	Canceled	*Passiflora incarnata* L./*Crataegus rhipidophylla* Gand.*/Digitalis* sp.
CARDIOSETYL M/simple herbal, simple anxiolytic	Oral solution	21.573.449.0001-19	Canceled	*Passiflora incarnata L.*
CARDIOSETYL M/herbal compound/antispasmodic	Oral solution	21.573.449/0001-19	Canceled	*Passiflora alata* Curtis/*Crataegus rhipidophylla* Gand./*Erythrina mulungu* Mart./ *Leptolobio elegans* Vog
DALAY/simple herbal	Coated tablet/oral solution	01.858.973/0001-29	Valid	*Passiflora* dry extract
DYRAJAIA/herbal compound	Tincture	02.007.074/0001-85	Canceled	*Passiflora alata* Curtis/*Dorstenia multiformis* Miq.
ELIXIR DE MARACUJÁ COMPOSTO FARMAERVAS/herbal compound	Elixir	60.565.520/0001-73	Canceled	*Passiflora alata* Curtis /*Paullinia cupana* Kunth/ *Valeriana officinalis* L.
EQUILIBRISSE/simple herbal, simple anxiolytic	Coated tablet	05.161.069/0001-10	Valid	*Passiflora* dry extract
EQUILIBRISSE/simple herbal, simple anxiolytic	Coated tablet	05.161.069/0001-10	Valid	*Passiflora incarnata L.*
ELIXIR DE CEREUS COMPOSTO/herbal compound	Oral solution	92.695.816/0001-03	Canceled	*Crataegus* tincture/*Cereus grandiflorus* (L.) Mill./*Valeriana officinalis* L./*Passiflora alata* Curtis
ERVA SILVINA COMPOSTA/herbal compound	Elixir	92.943.992/0001-09	Canceled	*Casearia sylvestris* Sw./*Aristolochia cymbifera* Martius/*Passiflora alata* Curtis
FIMATOSAN/Associated natural products	Oral solution	33.379.887/0001-96	Canceled	*Caesalpinia ferrea* Mart./*Roripa nasturtium* Beck/*Mikania glomerata* Spreng./*Passiflora alata* Curtis/*Polypodium vulgare*/*Myrospermum erythroxylum* Allem./*Lantana camara* Linné
FIQUEZEN/simple herbal, simple anxiolytic	Coated tablet/oral solution	17.875.154/0001-20	Valid	*Passiflora* dry extract
FITOCALM/simple herbal, simple anxiolytic	Coated tablet	84.684.620/0001-87	Valid	*Passiflora incarnata* L.
FITOCALMIN/simple herbal	Hard gelatin capsule	14.186.324/0001-70	Canceled	*Passiflora incarnata L.*
FLORINY/Anxiolytics—drug combination/herbal compound	Coated tablet/oral solution	64.088.172/0001-41	Canceled	*Passiflora incarnata* L./*Salix alba* L./*Crataegus oxyacantha* L.
MARACUGINA COMPOSTA/herbal compound/anxiolytic	Oral solution/coated tablet	61.082.426/0002-07	Canceled	*Erythrina mulungu* Mart./*Crataegus rhipidophylla* Gand*./Passiflora alata* Curtis
MARACUGINA COMPOSTA/herbal compound	Oral solution/coated tablet	67.866.665/0002-34	Canceled	*Passiflora alata* Curtis/*Erythrina mulungu* Mart./*Crataegus rhipidophylla* Gand.
MARACUGINA COMPOSTA/herbal compound	Oral solution/coated tablet	02.932.074/0001-91	Canceled	*Passiflora alata* Curtis/*Erythrina mulungu* Mart./*Crataegus rhipidophylla* Gand.
MARACUGINA PI/ simple herbal, simple anxiolytic	Coated tablet	61.082.426/0002-07	Valid	*Passiflora* dry extract
MARACUGINA PI/ simple herbal, simple anxiolytic	Coated tablet	61.082.426/0002-07	Valid	*Passiflora incarnata L.*
MARACUJÁ CONCENTRIX/herbal compound, simple anxiolytic	Simple dragees/oral solution	05.044.984/0001-26	Canceled	*Crataegus rhipidophylla* Gand./*Passiflora incarnata* L./*Salix alba* L.
MARACUJÁ GOTAS/simple herbal	Hydroalcoholic solution	78.950.011/0001-20	Canceled	*Passiflora incarnata L.*
MARACUJÁ HERBARIUM/simple herbal, simple anxiolytic	Hard gelatin capsule/coated tablet	78.950.011/0001-20	Valid	*Passiflora incarnata L.*
MULUNGU BROMETADO/similar	Syrup	10.419.935/0001-60	Canceled	KBr/NaBr/ NH4Br/*Atropa belladonna* tincture/*Hyosciamus niger* L. Tincture/*Passiflora alata* Curtis
NERVITON/Associated phytotherapics	Elixir	92.751.965/0001-34	Canceled	*Passiflora alata* Curtis/*Ptychopetalum olacides* Benth/*Cola nitida* Schott & Endl./*Paullinia cupana* Kunth/thiamine hydrochloride
NEUREXAN/Dynamized compound	Sublingual tablet	05.994.539/0001-27	Valid	*Avena sativa* L./ *Coffea arabica* L./ *Passiflora incarnata* L./*Zincum isovalerianicum*
NUIT/herbal compound	Coated tablet	49.475.833/0001-06	Canceled	*Passiflora incarnata* L./ *Valeriana officinalis* L./*Crataegus rhipidophylla* Gand.
PASALIX/herbal compound	Coated tablet	60.726.692/0001-81	Valid	C*rataegus rhipidophylla* Gand./*Passiflora incarnata* L./*Salix alba* L.
PASALIX PI/simple herbal, simple anxiolytic	Coated tablet/oral solution	60.726.692/0001-81	Valid	*Passiflora incarnata* L.
PASIC/herbal compound	Coated tablet/oral aerosol	44.734.671/0001-51	Canceled	*Passiflora incarnata* L./ *Crataegus rhipidophylla* Gand./*Salix alba* L.
PASSANEURO/herbal compound	Coated tablet	47.100.862/0001-50	Canceled	*Passiflora* dry extract/ *Erithrina mulungu* Mart./*Matricaria chamomilla* L.
PASSICALM/herbal compound	Oral solution	85.776.524/0001-21	Canceled	*Erithrina mulungu* Mart./*Valeriana officinalis* L./ *Passiflora alata* Curt
PASSIENE/simple herbal	Syrup	78.950.011/0001-20	Canceled	*Passiflora* dry extract
PASSIENE/simple herbal	Syrup/ coated tablet	78.950.011/0001-20	Valid	*Passiflora incarnata L.*
PASSIFLORA/herbal compound	Tincture	02.007.074/0001-85	Canceled	*Passiflora* dry extract/ *Erithrina mulungu* Mart.
PASSIFLORA ALTHAIA/simple herbal, simple anxiolytic	Oral solution	48.344.725/0007-19	Valid	*Passiflora incarnata L.*
PASSIFLORA CATARINENSE/simple herbal, simple anxiolytic	Coated tablet	84.684.620/0001-87	Valid	*Passiflora incarnata L.*
PASSIFLORA COMPOSTA/herbal compound	Oral solution/ simple dragee	42.341.149/0001-84	Canceled	*Crataegus rhipidophylla* Gand./*Passiflora incarnata* L./*Eerythrina mulungu* Mart.
PASSIFLORA COMPOSTA/herbal compound	Elixir	61.299.111/0001-35	Canceled	*Erythrina mulungu* Mart./*Melissa officinalis* L./*Passiflora alata* Curtis
PASSIFLORA DA ÍNDIA/associated natural products	Elixir	87.104.170/0001-02	Canceled	*Passiflora alata* Curtis*/ Atropa belladonna* L.*/ Erythrina mulungu* Mart./*Citrus limetta* Risso/*Cereus grandiflorus* (L.) Mill.
PASSIFLORA KLEIN/simple herbal, simple anxiolytic	Tincture	92.762.277/0001-70	Valid	*Passiflora incarnata L.*
PASSIFLORA ORIENT/simple herbal	Hard gelatin capsule	73.657.876/0001-89	Canceled	*Passiflora incarnata L.*
PASSIFLORINE/herbal compound	Oral solution/simple dragee	33.388.182/0001-79	Canceled	*Passiflora incarnata* L./*Salix alba* L./*Crataegus rhipidophyla* Gand./Calcium gluconate/Cholecalciferol/low molecular weight peptides/magnesium hyposulfium
PASSIFLORINE PI/simple herbal, simple anxiolytic	Coated tablet	23.668.196/0001-92	Valid	*Passiflora* dry extract
PASSIPAX/simple herbal	Oral solution	04.656.253/001-79	Valid	*Passiflora incarnata L.*
PAZINE/simple herbal, simple anxiolytic	Coated tablet	64.088.172/0001-41	Canceled	*Passiflora incarnata L.*
PAZINE/simple herbal, simple anxiolytic	Coated tablet	07.670.111/0001-54	Valid	*Passiflora incarnata L.*
PHYTOCALM /herbal compound	Syrup/hard gelatin capsule	84.684.620/0001-87	Canceled	*Passiflora incarnata* L./*Valeriana officinalis* L./*Matricaria chamomilla* L./*Crataegus oxyacantha* L./*Erythrina mulungu* Mart.
PRAKALMAR/Simple herbal, simple anxiolytic	Coated tablet	02.433.631/0001-20	Valid	*Passiflora incarnata L.*
PRASILENCE/Simple herbal, simple anxyolitic	Oral solution/coated tablet	25.773.037/0001-83	Valid	*Passiflora incarnata L.*
RITMONEURAN/Herbal compound	Hard gelatin capsule/oral solution	92.695.691/0001-03	Canceled	*Passiflora alata* Curtis/*Erythrina mulungu* Mart./*Leptolobio elegans* Vog/*Adonis vernalis* L.
RITMONEURAN RTM/simple anxiolytic, simple herbal	Hard gelatin capsule/oral solution/ coated tablet	92.695.691/0001-03	Valid	*Passiflora* dry extract
SALSAPARILHA COMPOSTO/Associated natural products	Elixir	92.943.992/0001-09	Canceled	*Smilax japicanga* Griseb./*Baccharis genistelloides* Persoon/*Plantago major* L./*Aristolochia cymbifera* Martius/*Passiflora incarnata* L.
SEAKALM/herbal compound	Coated tablet	02.456.955/0001-83	Canceled	*Passiflora incarnata* L./*Salix alba* L./*Crataegus rhipidophylla* Gand.
SEAKALM/simple anxiolytic, simple herbal	Coated tablet/oral solution	02.456.955/0001-83	Valid	*Passiflora incarnata L.*
SEDACAL/ simple herbal	Hard gelatina capsule	01.845.448/0001-79	Canceled	*Passiflora incarnata L.*
SEDANUS/associated natural products	Oral solution	67.866.665/0002-34	Canceled	*Crataegus oxyacantha* L./*Passiflora incarnata* L./*Erythrina mulungu* Mart.
SERENUS/associated phytotherapics	Coated tablet	49.475.833/0001-06	Valid	*Crataegus rhipidophylla* Gand./*Passiflora incarnata* L./*Salix alba* L.
SINTOCALMY/simple herbal simple anxiolytic	Coated tablet	60.659.463/0001-91	Valid	*Passiflora incarnata L.*
SOMINEX/herbal compound	Tablet	57.507.378/0001-01	Canceled	*Crataegus rhipidophylla* Gand./*Valeriana officinalis* L./*Passiflora incarnata* L.
SOMINEX COMPOSTO/herbal compound anxiolytics—drug combinations	Coated tablet	57.507.378/0001-01	Canceled	*Valeriana officinalis* L./*Crataegus rhipidophylla* Gand./ *Passiflora incarnata* L.
SONOTABS/herbal compound	Simple dragee	92.695.691/0001-03	Canceled	*Passiflora* dry extract/*Salix* dry extract/C*rataegus rhipidophylla* Gand. extract
SONOTABS/herbal compound	Simple dragee	90.455.262/0001-33	Canceled	*Passiflora* dry extract/*Salix* dry extract/C*rataegus rhipidophylla* Gand. extract
SPASCUPREEL/potentized compound	Sublingual tablet/injectable solution	05.994.539/0001-27	Valid	*Aconitum napellus* L./ Ammonium bromatum/*Agaricus muscarius*/*Citrullus colocynthis* Schrad./Colocynthis/*Gelsemium sempervirens* (L.) J. St.-Hil./ Magnesium phosphoricum/*Matricaria recutita* L./ *Passiflora incarnata* L./ *Veratrum album* L./ Atropinum sulfuricum/Cuprum suphuricum
SPASCUPREEL/potentized compound	Sublingual tablet/	74.455.197/0001-90	Canceled	Atropinum sulfuricum/*Citrullus colocynthis* Schrad./*Veratrum album* L./ Magnesium phosphoricum/*Gelsemium sempervirens* (L.) J. St.-Hil./ *Passiflora incarnata* L./ Agaricus muscarius/*Chamomilla recutita*/*Aconitum napellus*
TENSART/simple herbal	Coated tablet/oral solution	64.088.172/0001-41	Canceled	*Passiflora incarnata L.*
TENSART/ simple herbal	Coated tablet/oral solution	17.440.261/0001-25	Valid	*Passiflora incarnata L.*

Source: https://consultas.anvisa.gov.br/#/medicamentos/. Accessed on December 03, 2019.

## References

[1] Carlini E. A. (2003). Plants and the central nervous system. *Pharmacology Biochemistry and Behavior*.

[2] Dhawan K., Kumar S., Sharma A. (2002). Comparative anxiolytic activity profile of various preparations of *Passiflora incarnata* Linneaus: a comment on medicinal plants’ standardization. *Journal of Alternative and Complementary Medicine*.

[3] Wakdikar S. (2004). Global health care challenge: Indian experiences and new prescriptions. *Electronic Journal of Biotechnoly*.

[4] Germany, Federal Law Gazette [BGBl] (2005). Medicinal products act, part I. http://www.gesetze-im-internet.de/englisch_amg/medicinal_products_act.pdf.

[5] E Union (2001). *Directive 2001/83/EC*.

[6] E Union (2004). *Directive 2004/24/EC*.

[7] Miroddi M., Mannucci C., Mancari F., Navarra M., Calapai G. (2013). Research and development for botanical products in medicinals and food supplements market. *Evidence-Based Complementary Alternative Medicine*.

[8] Brazil, Ministry of Health (2010). *“RDC no 14/March 31, 2010,” Diário Oficial da União*.

[9] Brazil, Ministry of Health (2014). *“RDCN. 26/May 13, 2014,” Diário Oficial da União, Brasilia, Section 1*.

[10] Arifin S. F., Al Shami A., Omar S. S. S., Jalil M. A. A., Khalid K. A., Hadi H. (2019). Impact of modern technology on the development of natural-based products. *Journal of Ayurvedic and Herbal Medicine*.

[11] Scopus® S.V. (2019). *Bibliographic Database and Search Site*.

[12] Scirus® (2019). *Bibliographic Database and Search Site*.

[13] SciFinder® (2019). *Bibliographic Database and Search Site*.

[14] Google Scholar® (2019). *Search Site*.

[15] United States, Department of Health and Human Services (2020). *Database. ClinicalTrials.Gov.*.

[16] Brazil, National Institute of Industrial Property (INPI) (2019). *Search Website*.

[17] Brazil, Ministry of Health (2019). *Datavisa ANVISA Websitae*.

[18] World Trade Organization (1994). *Agreement on Trade-Related Aspects of Intellectual Property Rights*.

[19] Brazil, Ministry of Health (2019). *Brazilian Pharmacopoeia*.

[20] MHRA (Medicines and Healthcare Products Regulatory Agency) (2014). *Guidance: Herbal Medicines Granted a Traditional Herbal Registration*.

[21] HMPC (2014). *Assessment Report on Passiflora Incarnata L. Herba*.

[22] Muschner V. C., Lorenz A. P., Cervi A. C., Bonatto S. L., Souza-Chies T. T. (2003). A first molecular phylogenetic analysis of *Passiflora* (Passifloraceae). *American Journal of Botany*.

[23] Ulmer T., Mac Dougal J. M. (2004). *Passiflora: Passionflowers of the World*.

[24] Pereira C. A. M., Vilegas J. H. Y. (2000). Constituintes químicos e farmacologia do gênero *Passiflora* com ênfase a *P. alata* Dryander. *Revista Brasileira de Plantas Medicinais*.

[25] Souza M. M., Pereira T. N. S., Vieira M. C. (2008). Cytogenetic studies in some species of *Passiflora* L. (Passifloraceae): a review emphasizing Brazilian species. *Brazilian Archives of Biology Technology*.

[26] Cerqueira-Silva C. B. M., Conceição L. D. H. C. S., Souza A. P., Corrêa R. X. (2014). A history of passion fruit woodiness disease with emphasis on the current situation in Brazil and prospects for Brazilian passion fruit cultivation. *European Journal of Plant Pathology*.

[27] Gosmann G., Provesnsi G., Comunello L. N., Rates S. M. K. (2011). Composição química e aspectos farmacológicos de espécies de *Passiflora* L. (Passifloraceae). *Revista Brasileira de Biociencias*.

[28] Oliveira J. C., Ruggiero C., Faleiro F. G., Junqueira N. T. V., Braga M. F. (2005). Espécies de Maracujá com potencial agronômico,. *Maracujá germoplama e melhoramento genético*.

[29] Braga A., Medeiros T. P., Araújo B. V. (2010). Investigação da atividade antihiperglicemiante da farinha da casca de *Passiflora edulis* Sims, Passifloraceae, em ratos diabéticos induzidos por aloxano. *Revista Brasileira de Farmacognosia*.

[30] Miroddi M., Calapai G., Navarra M., Minciullo P. L., Gangemi S. (2013). *Passiflora incarnata* L.: ethnopharmacology, clinical application, safety and evaluation of clinical trials. *Journal of Ethnopharmacology*.

[31] Klein N., Gazola A. C., C de Lima T., Schenkel E., Nieber K., Butterweck V. (2014). Assessment of sedative effects of *Passiflora edulis* f. *flavicarpa* and *Passiflora alata* extracts in mice, measured by telemetry. *Phytotherapy Research*.

[32] Cervi A. C., Rodrigues W. A. (2010). Nomenclatural and taxonomic review of Passifloraceae species illustrated and described by Vellozo in Flora Fluminensis. *Acta Botanica Brasilica*.

[33] Dhawan K., Dhawan S., Sharma A. (2004). *Passiflora*: a review update. *Journal of Ethnopharmacololy*.

[34] De-Paris F., Petry R. D., Reginatto F. H. (2002). Pharmacochemical study of aqueous extract of *Passiflora alata* Dryander and *Passiflora edulis* Sims. *Acta Farmaceutica Bonaerense*.

[35] Soulimani R., Younos C., Jarmouni S., Bousta D., Misslin R., Mortier F. (1997). Behavioural effects of *Passiflora incarnata* L. and its indole alkaloid and flavonoid derivatives and maltol in the mouse. *Journal of Ethnopharmacology*.

[36] Oliveira M. S., Pinheiro I. O., Silva F. S. B. V. (2015). Vermicompost and arbuscular mycorrhizal fungi: an alternative to increase foliar orientin and vitexin-2-O-ramnoside synthesis in *Passiflora alata* Curtis seedlings. *Industrial Crops and Products*.

[37] Rehwald A., Meier B., Sticher O. (1994). Qualitative and quantitative reversed-phase high-performance liquid chromatography of flavonoids in *Passiflora incarnata* L. *Pharmaceutica Acta Helvetiae*.

[38] Gomes S. V. F., Portugal L. A., Anjos J. P. (2017). Accelerated solvent extraction of phenolic compounds exploiting a Box-Behnken design and quantification of five flavonoids by HPLC-DAD in *Passiflora* species. *Microchemical Journal*.

[39] Zucolotto S. M., Fagundes C., Reginatto F. H. (2012). Analysis of C-glycosyl flavonoids from south American *Passiflora* species by HPLC-DAD and HPLC-MS. *Phytochemical Analysis*.

[40] Ulubelen A., Oksuz S., Mabry T. J., Dellamonica G., Chopin J. (1982). C-glycosylflavonoids from *Passiflora pittieri, P. alata, P. ambigua* and *Adenia mannii*. *Journal of Natural Products*.

[41] Farag M. A., Otify A., Porzel A., Michel C. G., Elsayed A., Wessjohann L. A. (2016). Comparative metabolite profiling and fingerprinting of genus *Passiflora* leaves using a multiplex approach of UPLC-NS and NMR analysed by chemometric tools. *Analytical and Bioanalytical Chemistry*.

[42] Wosch L., Santos K. C., Imig D. C. (2017). Comparative study of *Passiflora* taxa leaves: II. A chromatographic profile. *Brazilian Journal Pharmacognosy*.

[43] Lin C. L., Kao C. L., Huang S. C. (2016). Chemical constituents of fruit shells of *Passiflora edulis*. *Chemistry Natural Compounds*.

[44] Hu Y., Jiao L., Jiang M. H. (2018). A new C-glycosyl flavone and a new neolignan glycoside from *Passiflora edulis* Sims peel. *Natural Products Research*.

[45] Petry R. D., De Souza K. C. B., Bassani V. L., Petrovick P. R., Ortega G. G. (1998). Doseamento do teor de flavonóides totais em extratos em extratos hidroalcóolicos de *Passiflora alata* Dryander (maracujá). *Revista Brasileira de Farmácia*.

[46] Petry R. D., Reginatto F., De-Paris F. (2001). Comparative pharmacological study of hydroethanol extracts of *Passiflora alata* and *Passiflora edulis* leaves. *Phytotherapy Research*.

[47] Müller S. D., Vasconcelos S. B., Coelho M., Biavatti M. V. (2005). LC and UV determination of flavonoids from: *Passiflora alata* medicinal extracts and leaves. *Journal of Pharmaceutical and Biomedical Analysis*.

[48] Pereira C. A. M., Yariwake J. H., Lanças F. M., Wauters J. N., Tits M., Angenot L. (2004). A HPTLC densitometric determination of flavonoids from *Passiflora alata, P. edulis, P. incarnata* and *P. caerulea* and comparison with HPLC method. *Phytochemical Analysis*.

[49] Reginatto F., De-Paris H. F., Petry R. D. (2006). Evaluation of anxiolytic activity of spray dried powders of two South Brazilian *Passiflora* species. *Phytotherapy Research*.

[50] Gonzáles Ortega G., Schmidt P. C. (1995). Stability studies on dried extracts of passion flower (*Passiflora incarnata* L.). *Pharma Sciences*.

[51] Quercia V., Turchetto L., Pierini N., Cuozzo V., Percaccio G. (1978). Identification and determination of vitexin and isovitexin in *Passiflora incarnata* extracts. *Journal of Chromatograph A*.

[52] Jay M., Harbone J. B. (1996). C-glycosylflavonoids. *The Flavonoids*.

[53] Wohlmuth H., Penman K. G., Pearson T., Lehmann R. P. (2010). Pharmacognosy and chemotypes of passionflower (*Passiflora incarnata* L.). *Biological and Pharmaceutical Bulletin*.

[54] Zeraik M. L., Pereira C. A. M., Zuin V. G., Yariwake J. H., Maracujá (2010). Um alimento funcional?. *Revista Brasileira de Farmacognosia*.

[55] Harbone J. B., Baxter H. (1995). *Phytochemical Dictionary: A Handbook of Bioactive Compound from: Plants*.

[56] Lutomski J., Wrocinski T. (1960). Pharmacodinamic properties of *P. incarnata* preparations. The effect of alkaloid and flavonoid components on pharmacodinamic properties of the raw materials. *Biuletyn Instytutu Roslin Leczniczych*.

[57] WHO, Passiflorae H. (2007). *WHO Monographs on Selected Medicinal Plants*.

[58] Wasowski C., Marder M. (2012). Flavonoids as GABA_A_ receptor ligands: the whole story?. *Journal of Experimental Pharmacology*.

[59] Wang F., Huen M. S. Y., Tsang S. Y., Xue H. (2005). Neuroactive flavonoids interacting with GABA_A_ receptor complex. *Current Drug Targets*.

[60] Zhang W., Koehler K. F., Zhang P., Cook J. M. (1995). Development of a comprehensive pharmacophore model for the benzodiazepine receptor. *Drug Design and Discovery*.

[61] Hanrahan J. R., Chebib M., Johnston G. A. R. (2011). Flavonoid modulation of GABA_A_ receptors. *British Journal of Pharmacology*.

[62] Dekermendjian K., Kahnberg P., Witt M. R., Sterner O., Nielsen M., Liljefors T. (1999). Structure–activity relationships and molecular modeling analysis of flavonoids binding to the benzodiazepine site of the rat brain GABA_A_ receptor complex. *Journal of Medicinal Chemistry*.

[63] Huang X., Liu T., Gu J. (2001). 3D-QSAR Model of flavonoids binding at benzodiazepine site in GABA_A_ receptors. *Journal of Medicinal Chemistry*.

[64] Kahnberg P., Lager E., Rosenberg C. (2002). Refinement and evaluation of a pharmacophore model for flavone derivatives binding to the benzodiazepine site of the GABA_A_ receptor. *Journal of Medicinal Chemistry*.

[65] Holanda D. K. R., Wurlitzer N. J., Dionisio A. P. (2020). Garlic passion fruit (*Passiflora tenuifila* Killip): assessment of eventual acute toxicity, anxiolytic, sedative, and anticonvulsant effects using *in vivo* assays. *Food Research International*.

[66] Zhang J., He Y., Jiang X., Jiang H., Shen J. (2019). Nature brings new avenues to the therapy of central nervous system diseases-an overview of possible treatments derived from natural products. *Science China Life Sciences*.

[67] Germán-Ponciano L. J., Puga-Olguín A., Rovirosa-Hernández M. J., Caba M., Meza E., Rodríguez-Landa J. F. (2020). Differential effects of acute and chronic treatment with the flavonoid chrysin on anxiety-like behavior and fos immunoreactivity in the lateral septal nucleus in rat. *Acta Pharmaceutica*.

[68] Alves J. S. F., Marques J. L., Demarque D. P. (2020). Involvement of isoorientin in the antidepressant bioactivity of a flavonoid-rich extract from *Passiflora edulis* f. *flavicarpa* leaves. *Revista Brasileira de Farmacognosia*.

[69] Rashno M., Ghaderi S., Nesari A., Khoesandi L., Farbood Y., Sarkaki A. (2020). Chrysin attenuates traumatic brain injury-induced recognition memory decline, and anxiety/depression-like behaviors in rats: insights into underlying mechanisms. *Psychopharmacology*.

[70] Appel K., Rose T., Fiebich B., Kammler T., Hoffmann C., Weiss G. (2011). Modulation of the *γ*-aminobutyric acid (GABA) system by *Passiflora incarnata* L. *Phytotherapy Research*.

[71] Viola H., Wasowski C., Stein M. L. (1995). Apigenin, a component of *Matricaria recutita* flowers, is a central benzodiazepine receptors-ligand with anxiolytic effects. *Planta Medica*.

[72] Vissiennona C., Nieber K., Kelber O., Butterweck V. (2012). Route of administration determines the anxiolytic activity of the flavonols kaempferol, quercetin and myricetin—are they prodrugs?. *The Journal of Nutritional Biochemistry*.

[73] Braestrup C., Nielsen M., Olsen C. E. (1980). Urinary and brain *β*-carboline-3-carboxylates as potent inhibitors of brain benzodiazepine receptors. *Proceedings of the National Academy of Sciences of the United States of America*.

[74] Bharate S. S., Mignani S., Vishwakarma R. A. (2018). Why are the majority of active compounds in the CNS domain natural products? A critical analysis. *Journal of Medicinal Chemistry*.

[75] Salerno S., Settimo F., Taliani S. (2012). Medicinal chemistry of indolylglyoxylamide GABA_A_/BzR high affinity ligands: identification of novel anxiolytic/non sedative agents. *Current Topics in Medicinal Chemistry*.

[76] Clayton T., Poe M. M., Rallapalli S. (2015). A review of the updated pharmacophore for the alpha 5 GABA(A) benzodiazepine receptor model. *International Journal of Medicinal Chemistry*.

[77] Brazil, Ministry of Health (2014). *“Normative instruction n. 2./May 13, 2014,” Diário Oficial da União, Brasilia, Section 1*.

[78] Brazil, Ministry of Health (2015). *Monografia da espécie Passiflora incarnata Linnaeus (maracujá-vermelho)*.

[79] Fierascu R. C., Fierascu I., Ortan A., Georgiew M. I., Sieniawska E. (2020). Innovative approaches for recovery of phytoconstituents from medicinal/aromatic plants and biotechnological production. *Molecules*.

[80] He S. M., Chan E., Zhou S. F. (2011). ADME Properties of herbal medicines in humans: evidence, challenges and strategies. *Current Pharmaceutical Design*.

[81] Huang C., Zheng C., Li Y., Wang Y., Lu A., Yang L. (2013). Systems pharmacology in drug discovery and therapeutic insight for herbal medicines. *Briefings in Bioinformatics*.

[82] Sut S., Baldan V., Faggian M., Peron G., Dall’Acqua S. (2016). Nutraceuticals, a new challenge for medicinal chemistry. *Current Medicinal Chemistry*.

[83] Rasool M., Malik A., Waquar S. (2018). In-Silico characterization and in-vivo validation of *Albizia* saponin-A, iso-orientin, and aalvadorin using a rat model of Alzheimer’s disease. *Frontiers in Pharmacology*.

[84] Wang M., Firrman J., Liu L., Yam K. (2019). A review on flavonoid apigenin: dietary intake, ADME, antimicrobial effects, and interactions with human gut microbiota. *BioMed Research International*.

[85] Mirza M. U., Ghori N.-Ul-H., Ikram N., Adil A. R., Manzoor S. (2015). Pharmacoinformatics approach for investigation of alternative potential hepatitis C virus nonstructural protein 5B inhibitors. *Drug Design, Development and Therapy*.

[86] Abraham M. H. (2004). The factors that influence permeation across the blood–brain barrier. *European Journal of Medicinal Chemistry*.

[87] Stamatovic S. M., Keep R. F., Andjelkovic A. V. (2008). Brain endothelial cell-cell junctions: how to “open” the blood brain barrier. *Current Neuropharmacology*.

[88] Ancuceanu R., Dinu M., Dinu-Pirvu C., Anuta V., Negulescu V. (2019). Pharmacokinetics of B-ring unsubstituted flavones. *Pharmaceutics*.

[89] Noh K., Oh D. G., Nepal M. R. (2016). Pharmacokinetic interaction of chrysin with caffeine in rats. *Biomolecules & Therapeutics*.

[90] Cicero A. F. G., Colletti A. (2018). Handbook of Nutraceuticals for Chemical Use. *Nutraceuticals Active on Central Nervous System*.

[91] Mori A., Hasegawa K., Murasaki M. (1993). Clinical evaluation of Passiflamin (*Passiflora* extract) on neurosis—multicenter double blind study in comparison with mexazolam. *Clinical Evaluation*.

[92] Miyasaka L. S., Atallah Á. N., Soares B. (2007). *“Passiflora for Anxiety Disorder,” Cochrane Database of Systematic Reviews Issue 1. Art. No CD004518*.

[93] Akhondzadeh S., Naghavi H. R., Vazirian M., Shayeganpour A., Khani M. (2001). Passionflower in the treatment of generalized anxiety: a pilot double-blind randomized controlled trial with oxazepam. *Journal of Clinical Pharmacy and Therapeutics*.

[94] Akhondzadeh S., Kashani L., Mobaseri M., Hosseini S. H., Nikzad S., Khani M. (2001). Passionflower in the treatment of opiates withdrawal: a double-blind randomized controlled trial. *Journal of Clinical Pharmacy and Therapeutics*.

[95] Movafegh A., Alizadeh R., Hajimohamadi F., Esfehani F., Nejatfar M. (2008). Preoperative oral *Passiflora incarnata* reduces anxiety in ambulatory surgery patients: a double-blind, placebo-controlled study. *Anesthesis & Analgesia*.

[96] Aslanargun P., Cuvas O., Dikmen B., Aslan E., Yuksel M. U. (2012). *Passiflora incarnata* Linneaus as an anxiolytic before spinal anesthesia. *Journal of Anesthesia*.

[97] Dantas L. P., Oliveira-Ribeiro A., Almeida-Souza L. M., Groppo F. C. (2017). Effects of *Passiflora incarnata* and midazolam for control of anxiety in patients undergoing dental extraction. *Medicina Oral, Patologia Oral y Cirurgia Bucal*.

[98] Ngan A., Conduit R. (2011). A double-blind, placebo-controlled investigation of the effects of *Passiflora incarnata* (passionflower) herbal tea on subjective sleep quality. *Phytotherapy Research*.

[99] Brazil, Presidency of the Republic (1996). *“Law No. 9.279/May 14, 1996,” Diário Oficial da União, Brasilia, Section 1*.

[100] Brazil, Presidency of the Republic (2006). *“Decree No 5.813/June 22, 2006,” Diário Oficial da União, Brasilia, Section 1*.

[101] Muller A. C., Macedo M. F. (2005). Patentes de fitomedicamentos: como garantir o compartilhamento dos benefícios de P & D e do uso sustentável de recursos genéticos. *Revista Fitos*.

[102] Brazil, Presidency of the Republic (1999). *“Law No 9.782/January 26, 1999,” Diário Oficial da União, Brasilia, Section 1*.

[103] Brazil, Ministry of Health (2014). *“RDC No 38/June 18, 2014,” Diário Oficial da União, Brasilia, Section 1*.

[104] Brazil, Ministry of Health (2014). *“RDC 66/November 26, 2014,” Diário Oficial da União, Brasilia, Section 1*.

[105] Brazil, Ministry of Health (2016). *“RDC 105/August 31, 2016,” Diário Oficial da União, Brasilia, Section 1*.

[106] United States (2019). Patent full-text databases. http://patft.uspto.gov/netahtml/PTO/search-bool.html.

